# Impact of involuntary out-patient commitment on reducing hospital services: 2-year follow-up

**DOI:** 10.1192/pb.bp.114.047464

**Published:** 2015-08

**Authors:** Laura Castells-Aulet, Miguel Hernández-Viadel, Jesús Jiménez-Martos, Carlos Cañete-Nicolás, Carmen Bellido-Rodríguez, Roman Calabuig-Crespo, Pedro Asensio-Pascual, Guillem Lera-Calatayud

**Affiliations:** 1 Benito Menni CASM, Barcelona; 2 University Clinic Hospital, Valencia; 3 Medical-Legal Institute of Valencia; 4 Doctor Peset University Hospital, Valencia; 5 Mental Health Center of Yecla, Murcia; 6 La Ribera Hospital, Valencia

## Abstract

**Aims and method** To evaluate whether involuntary out-patient commitment (OPC) in patients with severe mental disorder reduces their use of hospital services. This is a retrospective case-control study comparing a group of patients on OPC (*n* = 75) and a control group (*n* = 75) which was composed of patients whose sociodemographic variables and clinical characteristics were similar to those of the OPC group. Each control case is paired with an OPC case, so the control case must have an involuntary admission in the month that the index OPC case admission occurred. Emergency room visits, admissions and average length of hospital stay over a 2-year follow-up after the initiation of OPC were compared.

**Results** No statistically significant evidence was found in the use of mental healthcare services between the two groups. Different reasons for admission found between the groups limit similarity when comparing the two.

**Clinical implications** The findings cast doubt over the effectiveness of this legal measure to reduce emergency visits, the number of admissions and the length of stay in the hospital.

Involuntary out-patient treatment was introduced into North America and Australia in the early 1980s as a measure intended to benefit persons with severe mental disorder who need ongoing psychiatric care due to their poor adherence to treatment and lack of insight.^1^ Several publications confirm that adherence to pharmacological treatment in disorders such as schizophrenia diminishes relapses and, consequently, hospitalisations (revolving-door syndrome) and the progressive deterioration that successive recurrences bring about.^2-5^ In recent decades, there has been a gradual introduction of various forms of out-patient commitment (OPC). Nowadays, it is a reality in many countries, among them Australia, Israel, England, New Zealand and the USA.^6^ In Spain, although there is currently no specific legislation on OPC, it is used in some cities (e.g. Valencia, Alicante, Barcelona and San Sebastian).

Application of OPC has caused a sharp debate at both the legal and medical levels. Its defenders believe that it is a less restrictive measure than hospitalisation,^7-12^ ensures adherence to treatment facilitating clinical stability,^13^ and thus provides more freedom for patients. Opponents however believe that OPC does not respect human rights. It destroys the therapeutic relationship, discriminates against the psychiatric patient and increases their risk of stigmatisation.^14^ They base their argument on the lack of scientific evidence supporting the efficacy of OPC.

## OPC effectiveness data

Non-randomised studies have reported conflicting results. Some have found a statistically significant association with a reduced rate of admission^9,12,15-17^ and a reduced length of hospital stay.^9,12,15,16^ Swartz *et al*^9^ evaluated the effectiveness of the OPC programme in New York and found that while under OPC there is a reduction in the number of admissions and length of hospital stay. They also evaluated the perceptions of stigma, coercion and satisfaction with treatment during the OPC and found no changes. Once the OPC was terminated, there was a sustained improvement (lower rates of hospitalisation and medication non-adherence) in those patients who received intensive treatment or whose treatment lasted for more than 6 months. Another noteworthy result is that patients who were subject to an OPC combined with assertive community treatment (ACT) had a lower risk of hospitalisation than did those who received ACT alone, but the first group had greater resources at their disposal.

Van Dorn *et al*^10^ showed that reduced admission rates were maintained for 6 months after the OPC had ended. Nakhost *et al*^17^ evaluated the effectiveness of OPC in Canada, discovering an association between patients under an OPC and a reduction in the number of readmissions. In addition, they found evidence that this positive effect on the rate of hospitalisation remained after the OPC had ended. This was most notable in patients who had no admissions or who had only one admission prior to the implementation of the OPC. They found no association with the length of hospital stay. By contrast, other studies^18-20^ have shown an association between an increase in the rate of admissions and length of hospital stay.

In a recent randomised controlled trial (RCT) in the UK with 12 months' follow-up, Burns *et al*^21^ found no significant difference in the rate of admission and length of hospital stay in individuals under OPC. These results are consistent with two RCTs from the USA in which no significant differences were found in the use of health services, social functioning or quality of life between the OPC and treatment as usual.^22,23^ These three RCTs are the only ones we found in the literature on community treatment orders. Despite being in different jurisdictions with different mental health systems and different laws, the consistency of performance is significant.

A recent systematic review by Maughan *et al*^24^ which looked at effectiveness of OPC concluded that it has no significant effect on other outcomes of hospitalisation and use of community services. These results are consistent with previous reviews of international experience, for example by Churchill *et al*^25^ and Kisely *et al*.^6^

## Aims

Most previous studies have been performed in Anglo-Saxon countries so the aim of the present study is to provide information about the effectiveness of this legal measure to reduce the use of hospital services in other countries. Previous observational studies at Valencia (Spain) concluded that involuntary out-patient treatment might be useful for certain patients with severe mental disorder.^26^ Because of the weakness of observational studies, our team previously conducted a retrospective study of cases and controls during 1 year follow-up, but the results indicated that OPC wasn't more effective than standard treatment.^27^ We consider that the information offered by the present study can be of interest given the long period studied.

If one considers that OPC increases commitment to the achievement of clinical stability in patients with severe mental illness, then it is expected that the application of this legal measure will decrease both the number of emergency room visits and the number of hospital admissions, as well as shorten the length of hospital stay.

## Method

This is a retrospective study of cases and controls where we compare a group of patients under an OPC with a control group.

The study population consisted of all patients in the city of Valencia who had been under an OPC for at least 2 years at the time of the study's initiation in August 2009. The admission which prompted the request for OPC was considered the index admission.

The control group consisted of a sample of patients admitted to the psychiatric unit of the Hospital Clínico de Valencia. Each control case was paired with an OPC case, so each had to have an involuntary admission in the same month as the index OPC case. Moreover, the control case must have had the same clinical diagnosis, the same sociodemographic variables (age, gender, place of residence) and the same clinical characteristics (the same number of admissions during the 2 years before the index admission) as the paired OPC case. Both groups received a standard treatment consisting of out-patient psychiatric follow-up medication monitoring, and community-based treatment, such as day centres. The only difference between the two groups was that the control group was not under OPCs.

The study compared the number of psychiatric emergency visits, the number of admissions and length of stay in the hospital for the OPC and control groups over a 2-year follow-up once the OPC had been initiated.

We recorded the following for each patient: age, gender, place of residence, psychiatric diagnosis according to the DSM-IV-TR,^26^ number of psychiatric emergencies, number of hospital admissions, main reason for admission and average length of hospital stay for 2 years before and 2 years after the initiation of the OPC. Emergencies included psychiatric emergencies only. Admissions included all admissions, voluntary and involuntary, registered in the psychiatric services during the study period.

## Results

The two groups comprised 75 patients - 50 males (66.7%) and 25 females (33.3%) - each. The average age was 41.4 years for the OPC group and 41.7 years for the control group.

Diagnoses on Axis 1 are shown in Table 1. In both groups schizophrenia was the most common diagnosis, affecting approximately 3 out of 4 patients with OPC (73%). Bipolar disorder was the second most frequent diagnosis (12%), followed by schizoaffective disorder and delusional disorder.

**Table 1:** Diagnosis on Axis I (DSM-IV-TR)^23^

	*n (%)*	
	OPC group	Control group
Schizophrenia	55 (73)	57 (75)

Bipolar disorder	9 (12)	8 (11)

Schizoaffective disorder	6 (8)	5 (7)

Delusional disorder	5 (7)	5 (7)

Total sample	75 (100)	75 (100)

OPC, out-patient commitment.

There are differences in the motives for index admission between the groups (Table 2). In the involuntary OPC group the main reasons were clinical decompensation because of non-adherence to treatment (78%) and aggressive behaviour (22%). In the control group, admission occurred mostly due to clinical decompensation without a clear non-adherence to treatment (47%), for example inconsistent use of medication, changes in the pharmacological pattern or substance misuse.

**Table 2:** Main reason for index admission

	*n (%)*	
	OPC group	Control group
Aggressive behaviour	16 (22)	10 (16)

Abandonment of treatment	56 (78)	16 (26)

Suicide attempt	0	7 (11)

Clinical decompensation without clear treatment drop-out	0	29 (47)

Total	72 (100)	62 (100)

OPC, out-patient commitment.

The number of emergency visits, number of admissions and average length of hospital stay in the 2 years leading up to the start of the OPC index admission did not reach significant difference between the OPC group and the control group (Table 3). These results have led us to conclude that they were two ‘similar’ groups using healthcare services.

**Table 3:** Use of hospital mental health services in the 2 years before and in the 2 years during out-patient commitment (OPC)

	2 years before OPC, mean			2 years of OPC, mean		
	OPC group	Control group	*P*	OPC group	Control group	*P*
Admissions, *n*	2.16	2.4	0.6	1.69	1.34	0.4

Emergency visits, *n*	1.84	1.77	0.7	0.77	0.53	0.2

Hospital stay, days	21.94	21.33	0.7	16.41	19.55	0.3

Patients, *n* (%)	75 (100)	75 (100)		31 (41)	24 (32)	

Regarding the number of admissions in the 2 years before the index admission in both groups, more than half of the patients (52%) had one hospital admission (*n* = 39), 27% had two admissions (*n* = 20), and the rest (21%) had more than 2 admissions (between 3 and 6 in the previous 2 years).

At 2 years after the index admission, the same three variables were checked. A significant decrease in the number of emergency visits and admissions and a decrease in the average length of hospital stay were found in both the OPC and the control group when compared with the results for the previous 2 years. There were, however, no statistically significant differences found when comparing the OPC group with the control group (Table 3).

Overall, 41% of patients on an OPC and 32% of controls had a hospital admission during the 2 years after the index admission. Of the patients in the OPC group, 20% (*n* = 5) and of those in the control group 17% (*n* = 13) had only one admission, whereas the remainder (21% in the OPC group and 15% in the control group) had more than two admissions.

## Discussion

In the present work, and in agreement with the medical literature, OPC is applied with greater frequency to persons with schizophrenia (73%). Like other authors,^6,21,22,27-29^ we found no significant differences between the control and OPC groups in the use of healthcare services: number of emergency visits, number of hospital admissions and average length of hospital stay.

The results of this work cast a shadow of doubt on the effectiveness of OPC as a measure of compulsory treatment in the community. No statistically significant differences were found between the number of emergency visits (mean 0.77), admissions (mean 1.69) and the length of hospital stay (mean 16.41 days) for the OPC group and for the control group (0.53, 1.34 and 19.55 respectively) (Table 3). If efficacy is defined by the reduced use of hospital services as examined in this study, our results indicate that this legislation is not more effective than standard treatment.

### Limitations of the study

On the one hand, the differences found between the reasons for admission for the OPC group and for the control group undermine the similarity of the two study groups and, therefore, the suitability for comparison. On the other hand, there are the general limitations of retrospective case-control studies (such a study cannot rule out selection bias nor confounding bias given the impossibility of performing a random assignment of the participants).

It is striking that despite evidence in the literature that OPC has no significant effects on hospital service use outcomes, there is a remarkable consistency in the characteristics of patients who should undergo this intervention.^22,26^ The application of OPC should not be a generalised measure, but should be limited to those patients with severe mental disorders in whom a lack of therapeutic adherence will lead to a severe deterioration of the illness or the appearance of violent behaviour and, therefore, seriously compromise the patient's ability to live in the community.

One important question is whether OPC improves outcomes in services that are already offering a good quality of care. The answer at present appears to be no judging by the balance of evidence. Nevertheless, even if intensive follow-up programmes such as ACT are provided, it may be necessary in some cases to apply OPC.^30^

The effectiveness of OPC can be estimated using other outcomes, for example patient satisfaction or adherence to treatment during or after the application of OPC. Further studies are required to provide more information about the effectiveness of this treatment strategy and to clarify the contradiction between negative scientific evidence and its use in clinical practice.
